# Post-traumatic Carotid-Cavernous Fistula Following Penetrating Orbital Injury: A Case Report and Literature Review

**DOI:** 10.7759/cureus.102591

**Published:** 2026-01-29

**Authors:** David Camilo Gómez Cristancho, Jose David Suarez Mera, Gustavo Diaz, Bryan Gómez Cristancho, Juliana Molina Montañez, Jaime A Arias, Juan Sebastian Castro Sepúlveda, Luis Garcia Rairan

**Affiliations:** 1 Neurosurgery, National University of Colombia, Bogota, COL; 2 Pathology, Fundación Santa Fe de Bogotá, Bogota, COL; 3 Nutrition, Universidad El Bosque, Bogota, COL; 4 Epidemiology, Universidad del Rosario, Bogota, COL; 5 Medicine, Universidad El Bosque, Bogota, COL

**Keywords:** carotid-cavernous sinus fistula, endovascular treatment (evt), penetrating ocular trauma, stab wounds, traumatic vascular injury

## Abstract

Posttraumatic carotid-cavernous fistulas (CCFs) are rare but potentially sight-threatening vascular lesions, most commonly associated with high-energy craniofacial trauma. Direct high-flow CCFs following penetrating orbital injury are exceptionally uncommon and may present with delayed or misleading clinical features. This report describes the case of a 35-year-old homeless man who presented with a two-month history of progressive left orbital symptoms after a knife-inflicted injury. Initial treatment targeted a presumed infectious process; however, persistent proptosis, ocular pain, mydriasis, complete visual loss, and ophthalmoplegia prompted further evaluation. CT angiography, performed due to a contraindication to MRI, demonstrated a direct high-flow left CCF with marked dilation of the cavernous sinus and superior ophthalmic vein. Digital subtraction angiography confirmed a Barrow type A CCF. The patient underwent staged endovascular therapy. Initial transarterial coil embolization achieved approximately 80% occlusion, with partial improvement in proptosis but persistent visual impairment. Follow-up angiography revealed residual shunting, leading to a second-stage covered stent angioplasty. Despite this, residual flow persisted, and a third endovascular procedure was planned. The third-stage intervention was canceled because of inadequate social support, and the patient was discharged prematurely and lost to follow-up. CCFs are rare entities but should be strongly suspected in cases of penetrating ocular trauma associated with proptosis, chemosis, and orbital bruits. One of the most important determinants of visual prognosis is timely diagnosis and prompt management of these lesions. In the present case, the patient’s social vulnerability contributed to delayed recognition and treatment, resulting in complete loss of vision. Furthermore, the patient’s social circumstances directly limited completion of the planned therapeutic strategy. Thus, social vulnerability emerged as a key determinant of prognosis, influencing both clinical outcomes and access to definitive care.

## Introduction

Carotid-cavernous fistulas (CCFs) are abnormal arteriovenous communications between the carotid arterial system and the cavernous sinus (CS) [1]. CCF is an infrequent condition, observed in approximately 0.2% of patients who sustain craniocerebral trauma and in up to 3.8% of those with fractures of the skull base [2]. Approximately 75% of CCFs are traumatic, most commonly associated with skull base fractures following closed head injury. Less frequently, they arise after penetrating trauma, such as projectile or stab wounds that directly lacerate the cavernous segment of the internal carotid artery (ICA), resulting in a high-flow shunt into the CS. Iatrogenic causes, including carotid endarterectomy, endovascular procedures, and paranasal sinus surgery, account for a smaller proportion of cases [1].

From an angiographic standpoint, CCFs are classified using the Barrow system into direct fistulas (type A), characterized by a direct connection between the ICA and the CS, and indirect fistulas (types B-D), which involve dural branches of the ICA and/or external carotid artery [3-5]. Direct CCFs are typically traumatic and high-flow, whereas indirect fistulas are more often spontaneous and low-flow.

The diversion of high-pressure arterial blood into the CS leads to venous hypertension and retrograde venous drainage, most commonly through the superior and inferior ophthalmic veins and, less frequently, via cortical venous pathways. This pathophysiology underlies the characteristic clinical manifestations of CCFs, including proptosis, chemosis, cranial nerve palsies, visual impairment, and pulsatile tinnitus; in severe cases, cortical venous reflux may result in intracranial hemorrhage or elevated intracranial pressure [6].

The primary goal of treatment is complete occlusion of the fistulous communication to relieve venous hypertension and prevent progressive visual or neurological deterioration. Visual recovery and symptom resolution depend largely on the chronicity of the fistula and the timing of intervention [7].

Although traumatic CCFs most commonly follow closed head injury or penetrating craniofacial trauma [8], those arising specifically from orbital stab wounds are exceedingly rare. The case presented in this report is clinically significant as it illustrates an uncommon mechanism, penetrating orbital injury, resulting in a direct, high-flow Barrow type A CCF with delayed symptom onset and management challenges requiring staged endovascular therapy. Notably, the case highlights how delayed diagnosis and intervention, compounded by the patient’s social vulnerability, contributed to poor visual outcomes and adversely affected the potential for functional recovery.

## Case presentation

A 35-year-old homeless man with no known comorbidities presented with a two-month history of progressive left orbital symptoms following a knife-inflicted penetrating injury. Initial manifestations included periorbital ecchymosis, edema, and purulent conjunctival discharge. The injury was initially managed as an infectious process with topical antibiotics. However, due to persistent symptoms, progressive ocular pain, and worsening visual impairment, the patient sought further medical evaluation.

**Figure 1:**
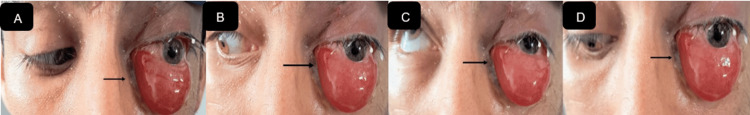
Ocular movements are demonstrated: (A) leftward gaze, (B) rightward gaze, (C) upward gaze, and (D) downward gaze. Arrows highlight the fixed position of the left eye, indicating a "frozen" appearance.

On examination, vital signs were stable. Neurological evaluation revealed no focal deficits other than the ocular findings. Ophthalmologic assessment demonstrated marked left-sided proptosis, periorbital ecchymosis, fixed mydriasis with absence of both direct and consensual light reflexes, and complete ophthalmoplegia of the left eye (“frozen eye”), consistent with paralysis of the third, fourth, and sixth cranial nerves. The right eye showed preserved visual acuity (20/20). The left eye had no light perception, suggesting involvement of the optic nerve (cranial nerve II). Intraocular pressure and the presence of an orbital bruit were not documented (Figure 1).

**Figure 2:**
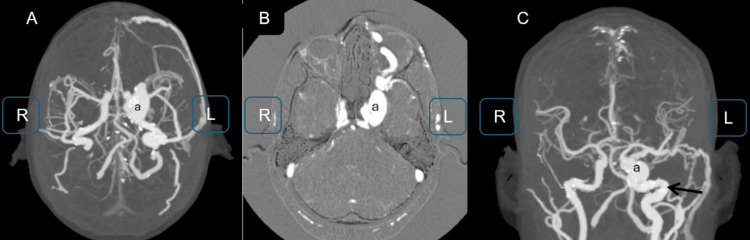
CTA with 3D and maximum intensity projection reconstructions: (A, B) axial images demonstrating a direct, high-flow CCF (a), characterized by early opacification and enlargement of the left CS in direct communication with the left ICA (arrow); (C) Coronal projection highlighting the fistulous point (a) and associated abnormal venous drainage, consistent with a Barrow type A fistula 3D: three-dimensional; CTA: computed tomography angiography; L: left; R: right; CCF: carotid-cavernous fistula; CS: cavernous sinus

Given the presence of retained metallic fragments in the frontal region, MRI was contraindicated. CTA revealed a direct left CCF with marked dilation of the CS, superior ophthalmic vein, and extraocular muscles, along with inflammatory changes in the periorbital soft tissues (Figure 2). No cortical venous reflux was identified on CTA.

Antibiotic therapy with a fourth-generation cephalosporin was administered for a four-week course due to the high risk of neuroinfection associated with a penetrating traumatic wound caused by a sharp object, with particular concern for gram-negative pathogens, in accordance with the infectious disease specialist’s recommendations. Following completion of antibiotic therapy, endovascular treatment was undertaken, confirming a left-sided Barrow type A CCF. The lesion was embolized via a transarterial approach using 11 coils of varying sizes, achieving approximately 80% obliteration of the fistulous connection.

**Figure 3:**
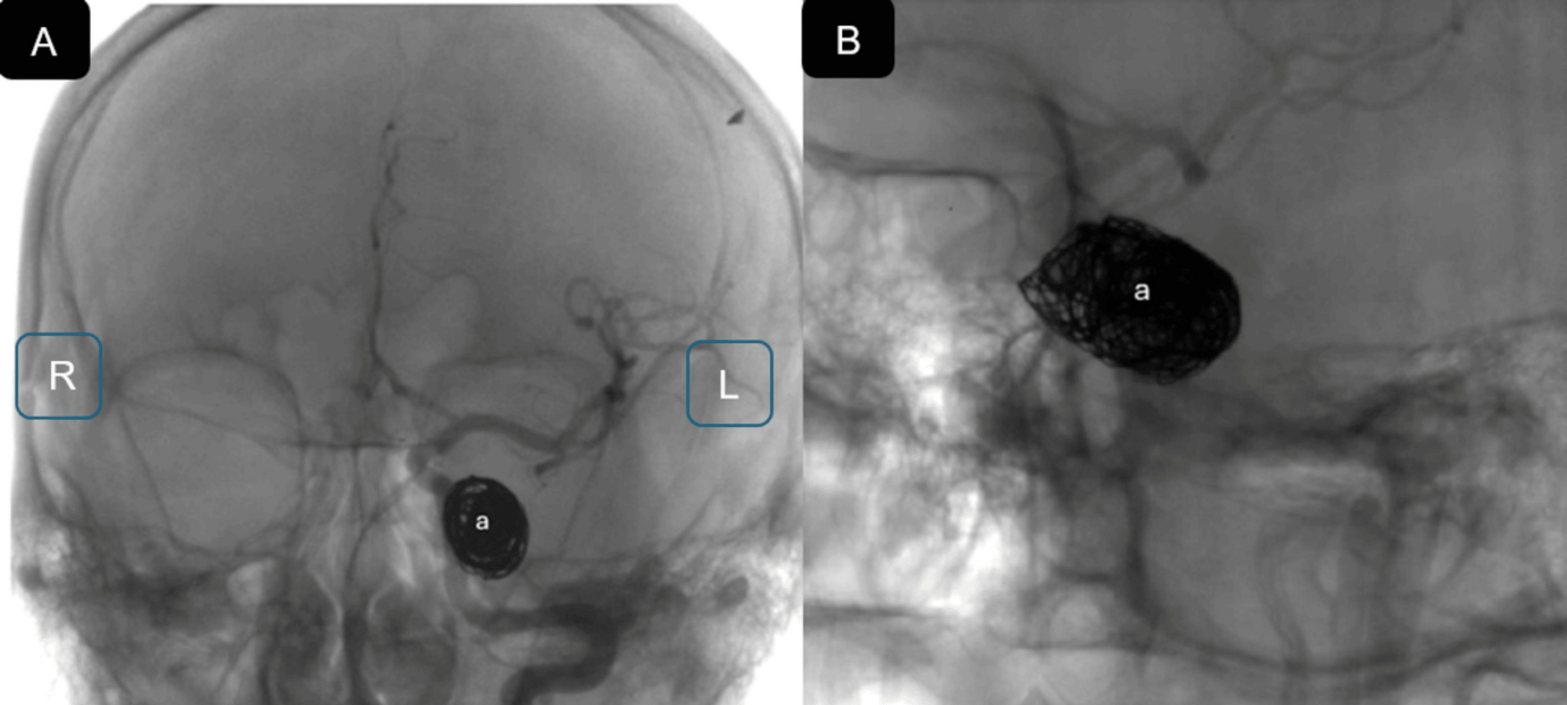
(A) Anteroposterior projection of a cerebral angiogram demonstrating approximately 80% embolization of the carotid–cavernous fistula (a); (B) Corresponding lateral of left side projection of the same partially embolized fistula (a) L: left; R: right

Angiographically, the CCF demonstrated a high-flow Barrow type A pattern with predominant anterior venous drainage through the superior ophthalmic vein, without evidence of dangerous cortical venous reflux at that stage. A focal pseudoaneurysmal component at the site of arterial injury was identified, supporting a staged endovascular strategy. Coil embolization was selected as the initial approach to reduce shunt flow, decrease venous hypertension, and stabilize the lesion while preserving patency of the supraclinoid segment of the left ICA. This resulted in a marked reduction of retrograde flow through the superior ophthalmic vein and maintained antegrade ICA flow (Figure 3).

Clinically, immediate post-procedural follow-up showed a significant reduction in left-sided proptosis, consistent with decreased orbital venous congestion. However, visual function did not improve, with persistent absence of light perception, and complete left ophthalmoplegia (“frozen eye”) remained unchanged, reflecting irreversible optic nerve injury and sustained cranial nerve III, IV, and VI dysfunction.

Fifteen days after the initial intervention, follow-up panangiography revealed persistent residual flow through the CCF, related to incomplete exclusion of the arterial defect and ongoing filling of the CS. Given the residual high-flow shunt and the presence of a pseudoaneurysmal arterial segment, a second-stage endovascular procedure was planned. This consisted of stent angioplasty using a covered stent (VIABAHN®; W. L. Gore & Associates, Inc., Newark, Delaware, United States), aimed at definitive reconstruction of the ICA and exclusion of the fistulous communication. The patient received dual antiplatelet therapy for one week prior to the procedure in accordance with endovascular stenting protocols.

The second-stage intervention was technically successful; however, post-procedural angiography demonstrated persistent residual fistulous flow, indicating incomplete sealing of the defect and justifying the need for a third-stage endovascular treatment to achieve complete fistula closure. Unfortunately, this procedure could not be performed due to the absence of adequate social support, specifically the lack of a family member to accompany the patient during hospitalization. The patient was discharged 72 hours after cancellation of the planned intervention and was subsequently lost to follow-up as a result of social vulnerability and insufficient family support.

## Discussion

What do we know about CCFs?

CCFs are abnormal arteriovenous communications between the carotid arterial system and the venous channels of the CS. Although CCFs secondary to craniomaxillofacial trauma have an incidence of less than 0.2%, they constitute the most frequent post-traumatic intracranial vascular anomaly [8].

Venous congestion within and surrounding the CS leads to elevated pressure throughout the regional venous system. The orbits, which drain into the CS through the superior and inferior ophthalmic veins, are typically the first structures to exhibit clinical manifestations of this retrograde venous flow. Depending on the fistula subtype, symptoms may appear within hours following injury in direct CCFs or develop insidiously over weeks to months in indirect CCFs [8].

Clinical manifestations

The clinical presentation of CCFs is variable and depends on the fistula type, flow dynamics, and venous drainage pattern. Proptosis is the most frequently reported manifestation, occurring in 72-98% of cases, followed by chemosis (55-100%), orbital bruits (71-80%), and headache (25-84%). Visual disturbances are common and include diplopia in up to 88% of patients, blurred vision, and orbital pain [5]. In the present case, clinical manifestations were predominantly ocular, with proptosis and complete ophthalmoplegia as the leading features, findings that are consistent with the most commonly reported presentations in the literature.

Additional manifestations include ophthalmoplegia, most commonly involving cranial nerve VI, which is particularly vulnerable due to its medial location within the CS. Other reported findings include pulsatile exophthalmos, ptosis, elevated intraocular pressure, anterior segment ischemia, papilledema, optic nerve atrophy, and involvement of the trigeminal nerve. The classic clinical triad associated with anterior CCFs consists of proptosis, bruit, and chemosis [8]. In contrast, posterior CCFs often lack these hallmark features and may present solely with ophthalmoplegia, making diagnosis more challenging.

The presence of an orbital bruit on auscultation is attributed to turbulent drainage of arterialized blood into the superior ophthalmic vein and may be accompanied by a palpable pulsatile proptosis. Although this finding is typically unilateral, bilateral involvement can occur in advanced cases. Visual deterioration results primarily from ischemic injury secondary to sustained elevation of intraocular pressure, leading to papilledema and secondary glaucoma [9]. Neuro-ophthalmologic manifestations in CCFs are driven by three principal pathophysiological mechanisms: elevated pressure within the CS, reversal of venous drainage toward the ophthalmic veins, and progressive intraocular hypertension affecting ocular structures [10]. In the present case, the patient’s predominant symptoms were ophthalmologic, including ecchymosis, periorbital edema, purulent conjunctival discharge, and ocular pain.

Diagnosis and classification

A high index of clinical suspicion is essential for the timely diagnosis of CCFs. While clinical features provide important diagnostic clues, imaging studies are critical for confirmation and characterization. Noninvasive imaging modalities, including CT, MRI, and CT or magnetic resonance angiography, are useful initial diagnostic tools. These studies may demonstrate indirect signs suggestive of CCF, such as enlargement of the CS, proptosis, extraocular muscle hypertrophy, dilation of the superior ophthalmic vein, engorgement of cortical or leptomeningeal veins, and associated craniofacial fractures.

Despite these advances, digital subtraction angiography (DSA) remains the gold standard for diagnosis, as it allows precise delineation of the fistula’s location, size, flow characteristics, venous drainage pattern, and potential herniation of the CS into adjacent structures [10].

CCFs can be classified according to etiology (traumatic or spontaneous), hemodynamic profile (high-flow or low-flow), and angioarchitecture. Angioarchitecturally, CCFs are divided into direct fistulas involving a direct connection between the ICA and the CS, and indirect dural arteriovenous fistulas involving meningeal branches of the ICA and/or the external carotid artery (ECA). The most widely adopted classification system is the Barrow classification, which categorizes CCFs as follows: (i) Type A fistulas, which represent a direct communication between the ICA and the CS; (ii) Type B fistulas, which involve meningeal branches of the ICA; (iii) Type C fistulas, which involve meningeal branches of the ECA; and (iv) Type D fistulas, which involve meningeal branches from both the ICA and ECA [11].

Type A fistulas are the most common and predominantly affect males between 12 and 46 years of age. Approximately 75% are traumatic, most frequently resulting from motor vehicle collisions, closed head injuries with basilar skull fractures, or penetrating trauma. These mechanisms produce laceration of the cavernous segment of the ICA, resulting in a high-flow arteriovenous shunt. Notably, cases of CCFs secondary to stab wounds involving the orbital region remain exceedingly rare in the literature [5].

Treatment

Although surgical intervention represents the most invasive therapeutic option for CCFs, it remains a definitive treatment modality in select cases. Reported success rates range from 31% to 79%, depending on the surgical technique and fistula characteristics [5]. Surgical strategies include direct suturing or clipping of the fistulous tract, packing of the CS, and ligation of the ICA [12].

Currently, endovascular therapy is considered the first-line treatment for CCFs, with reported cure rates exceeding 80% in most contemporary series [3]. In direct, high-flow CCFs, a transarterial approach is generally preferred. After accessing the ICA, embolization may be achieved using detachable coils, liquid embolic agents, or a combination of both [12].

For indirect CCFs, the transvenous approach is favored due to the technical difficulty and increased risk of ischemic complications associated with transarterial catheterization of small meningeal feeders. The inferior petrosal sinus (IPS) is the most commonly utilized venous access route. When IPS access is not feasible, alternative venous pathways, including the facial vein or superior ophthalmic vein, may be employed [3].

Clinical outcomes vary depending on fistula type, associated complications, and timing of intervention. While most patients experience symptomatic improvement or complete resolution, some develop permanent neurological deficits or die, particularly in cases involving high-flow shunting or delayed diagnosis. Visual recovery is influenced by several factors, including fistula hemodynamics, promptness of treatment, and the extent of ischemic damage to the optic nerve or retina. Although recurrence is uncommon, post-treatment angiographic follow-up is strongly recommended to confirm complete obliteration of the fistulous connection [5].

Literature review

Methods

Literature search: A comprehensive literature review was conducted to identify relevant publications addressing CCFs, with particular emphasis on cases secondary to penetrating craniofacial trauma, especially those involving sharp or pointed objects. The aim was to compile clinical, radiological, and complications that could provide context and support for the case described.

Search strategy: The literature search was conducted using multiple electronic databases, including PubMed, Scopus, and Google Scholar. The search strategy combined the following keywords: "carotid-cavernous fistula" AND ("penetrating trauma" OR "sharp weapon injury" OR "cavernous sinus thrombosis"). No time restrictions were applied to the search.

Eligibility criteria: Peer-reviewed original articles, case reports, and case series that specifically addressed CCFs, with particular emphasis on those resulting from penetrating injuries, were included. Publications were included if written in English or Spanish. Studies were excluded if they met any of the following criteria: duplicate publications, case reports with poorly documented clinical information, review articles, conference or congress abstracts, commentaries, letters to the editor, or unpublished data and cases with findings suggestive of CCF but lacking angiographic confirmation.

Data extraction: Following the inclusion and exclusion criteria, relevant data from full-text articles were manually extracted, duplicates were removed, and entries were recorded in a dedicated database. A customized Excel spreadsheet (Microsoft Corporation, Redmond, Washington, United States) was used to collect the following variables: (i) Author; (ii) Country of origin and year of publication; (iii) Underlying etiology; (iv) Affected eye; (v) Symptoms; (vi) Signs; (vii) Imaging findings; (viii) Treatment; and (ix) Complications.

Results

**Figure 4:**
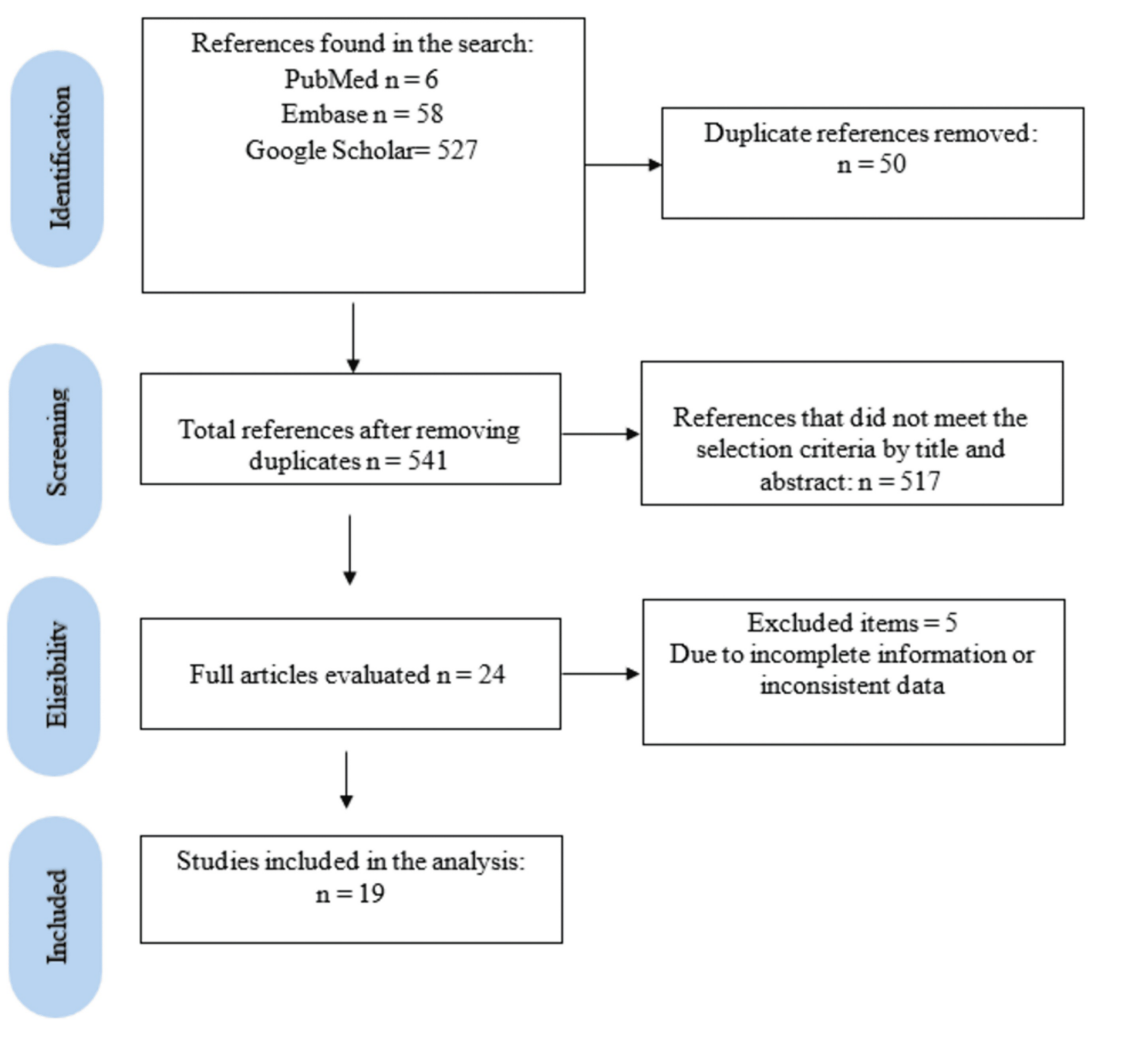
Flow diagram showing a selection of articles.

Among the 19 articles included in this review, most reported a single case; however, three articles described more than one case, yielding a total of 25 cases for analysis (Figure 4, Table 1). The most common etiologies of carotid-cavernous fistula (Table 2) were sharp object trauma and gunshot injuries, accounting for approximately 40% and 28% of cases, respectively. Blunt trauma was reported in 10% of cases, whereas less frequent mechanisms, each representing approximately 5%, included grenade fragment injuries, nail gun wounds, needlefish penetration, tree branch impalement, and wooden pencil injuries. All cases corresponded to high-flow fistulas, as detailed in Table 1.

**Table 1:** Summary of studies on carotid-cavernous fistula CCF: carotid–cavernous fistula; DSA: digital subtraction angiography; MRI: magnetic resonance imaging; MRA: magnetic resonance angiography; CTA: computed tomography angiography; ICA: internal carotid artery; CS: cavernous sinus

No.	Author	Country, year	Age (year), Sex	Etiology	Eye (side)	Symptoms	Imaging and findings	Acute complications	Treatment
1	Jelsma and Leaver [13]	United States, 1969	19, male	Grenade fragment wound	Right	Headache, eye protrusion, redness in conjunctiva	DSA: right internal, high-flow CCF	Acute cranial nerve paralysis, Horner syndrome	A right temporo-frontal craniotomy was performed. The right internal carotid and ophthalmic arteries were clipped, muscle fragments inserted via arteriotomy, and the carotid bulb resected. The right common, internal, and external carotid arteries were then ligated
2	Zimmer et al. [14]	United States, 1979	25, male	Gunshot wound	Right	Stuporous	DSA: Right internal carotid artery occlusion and a high-flow CCF	Hemiparesis, Died	Surgical intervention was performed, although the specific technique was not described
3	Kieck and de Villiers [15]	South Africa, 1984	Not specified; five cases were reported	Sharp object trauma	Right	CCF	DSA: High-flow CCF in right side	Not reported	ICA embolization
4	Bullock and van Dellen [16]	South Africa, 1985	24, male	Sharp object trauma	Left	Headache, facial pain	CTA: Acute high-flow CCF	Blind, ptosis	A right temporo-frontal craniotomy was performed, followed by occlusion of the right ICA and ophthalmic artery, insertion of muscle fragments via arteriotomy, resection of the carotid bulb, and ligation of the right common, internal, and external carotid arteries
5	Sherman and Gottlieb [17].	United States, 1988	26, male	Gunshot wound	Unclear	Not reported	CTA: High-flow CCF	Not reported	Embolization using a detachable balloon catheter
6	Khalil et al. [18]	Egypt and United States, 1991	12, male; 79, male	Blunt force trauma, Sharp object trauma	Right, Left	GCS 13/15, Periorbital swelling, eye swollen	DSA: Right high-flow CCF; DSA: Left high-flow CCF	Acute cranial nerve paralysis, proptosis; None	Embolization
									A detachable balloon was deployed in the left ICA to achieve complete fistula occlusion, and a Crutchfield clamp was gradually tightened on the common carotid artery over three days
7	Siniluoto et al. [19]	Finland, 1996	26, female	Blunt force trauma	Unclear	Not reported	DSA: high-flow CCF	Not reported	Transarterial embolization was performed with the deployment of four coils, resulting in initial partial occlusion; however, follow-up imaging confirmed complete exclusion of the CCF
8	Nayduch et al. [20]	United States, 2000	17, male	Tree branch	Left	GCS 6	CTA: Left high-flow CCF subarachnoid bleed, left hemispheric cerebellar hemorrhage	Hydrocephalus	A catheter inserted via the right femoral artery revealed a left Type A CCF. Multiple coils and a Detachable balloon were deployed, achieving complete occlusion with adequate crossflow from the right circulation
9	Gocmen et al. [21]	Turkey, 2012	46, male	Sharp object trauma	Left	Orbital bruit	DSA: High-flow CCF	None	Embolization
10	Yang et al. [22]	Canada, 2015	6, male	Sharp object trauma	Left	nausea, vomiting, pain, swelling, orbital bruit	MRA: Left high-flow CCF	Proptosis	The CCF was treated via a transvenous approach through the left facial, angular, and superior ophthalmic veins. Simultaneously, the ipsilateral ICA was catheterized to allow control angiography and roadmap guidance. A total of 16 detachable coils, including hydrogel-coated and bare platinum variants, were deployed within the left CS plexus to achieve embolization.
11	Alagöz et al. [23]	Turkey, 2016	26, male	Gunshot wound	Right	pain, redness, blurred vision, loss of vision	MRA: Right high-flow CCF	Not reported	Endovascular treatment was successfully performed via femoral artery
12	Marcellino et al. |[24]	United States, 2017	9, male	Gunshot wound	Unclear	closed eye diplopia, nausea, vomiting	DSA: Small high-flow CCF	Not reported	The patient was managed expectantly
13	Morais, et al. [25]	Brazil, 2017	8, male	Tree branch	left	nausea, vomiting, eye pain, eyelid swelling, hyperemia, double vision, mild orbital pain, headache	MRA: Direct left high-flow CCF	Not reported	Endovascular treatment was performed using a detachable balloon. Follow-up angiography demonstrated complete occlusion of the fistula with preservation of distal ICA flow.
14	Brooks et al. [26]	Australia, 2021	50, female	Nail gun wound	Unclear	Not reported	DSA: Direct high-flow CCF	Hydrocephalus, acute cranial nerve paralysis	The pseudoaneurysm was treated with coil embolization, and a stent was placed in the right ICA
15	Torche et al. [27]	Chile, 2021	6, female	Wooden pencil	Left	Not reported	CTA: High-flow CCF	None	Endonasal endoscopic access allowed visualization and removal of a transsellar foreign body extending to the right CS. Endovascular balloon control was used before extraction near the carotid artery. A CSF fistula was observed and repaired using fat graft, nasoseptal flap, and DuraSeal
16	Luo et al. [28]	China, 2022	50, female	Sharp object trauma	Left	Unconscious, vomiting GCS 11, eye swelling	CTA: High-flow CCF	persisting CCF pontine abscess blurred vision	Temporary ICA occlusion with a balloon was performed during foreign body removal; due to proximal and distal lacerations, the balloon remained inflated. The CCF and arterial lacerations were successfully occluded with coils
17	Hamedani et al. [29]	United States, 2022	Middle-aged	Gunshot wound	Left	Not reported	DSA: High-flow CCF	Mydriasis	Endovascular coilin
18	Loggini et al. [30]	United States, 2022	23, female; 30, male	Gunshot wound	Right left	Not reported	CTA: High flow CCF	mydriasis, pseudoaneurysm, hydrocephalus, CSF leak	The patient underwent emergent decompressive hemicraniectomy with placement of a right subgaleal drain. The traumatic CCF was embolized via a transvenous approach, achieving significant flow reduction. A flow-diverting stent was then deployed across the cavernous segment, further reducing arteriovenous shunting
									Initial balloon test occlusion was aborted due to loss of left hemispheric function. A flow-diverting stent improved perfusion, enabling a successful repeat test. The left ICA was then embolized, fully occluding the CCF
19	Alfaheed et al. [31]	Canada, 2023	17, female	Needlefish	Right	periorbital swelling, conjunctival chemosis, eyelid laceration	MRA: High-flow CCF	Acute cranial nerve paralysis, decreased visual acuity, ophthalmoplegia	Embolization

**Table 2:** Frequency of etiology of CCF in studies reviewed CCF: carotid-cavernous fistula References: [13-31]

Etiology	Frequency	Percentage
Gunshot wound	7	28%
Sharp object trauma	10	40%
Blunt force trauma	2	8%
Tree branch	2	8%
Grenade fragment wound	1	4%
Nail gun wound	1	4%
Needlefish	1	4%
Wooden pencil	1	4%
Total	25	100%

Analysis of clinical outcomes among the 25 patients included in this review revealed a wide spectrum of complications and prognoses (Table 3). Notably, outcome data were not reported in 40% of cases, limiting the ability to comprehensively assess morbidity across the full cohort. Among the remaining patients, the most frequently documented complication was cranial nerve paralysis, observed in 16% of cases.

**Table 3:** Frequency of outcomes in the studies reviewed CSF: cerebrospinal fluid; CCF: carotid-cavernous fistula References: [13-31]

Outcomes	Frequency	Percentage
Not reported	10	40%
Cranial nerve paralysis	4	16%
None	3	12%
Hydrocephalus	3	12%
Decreased visual acuity	2	8%
Proptosis	2	8%
CSF leak	1	4%
Died	1	4%
Hemiparesis	1	4%
Horner syndrome	1	4%
Persisting CCF	1	4%
Pontine abscess	1	4%
Ptosis	1	4%

The most frequently observed presenting symptoms are proptosis, ocular pain, visual impairment, and orbital bruit. A subset of patients, however, exhibits systemic manifestations such as vomiting or an altered level of consciousness, most often attributable to associated intracranial injuries.

Of the patients in the reviewed studies, 12% were reported to have no complications following treatment, while hydrocephalus was documented in another 12%. Decreased visual acuity and proptosis were reported in 8% of cases. Less frequent but clinically significant outcomes included cerebrospinal fluid leak, death, hemiparesis, Horner syndrome, persistent CCF, pontine abscess, and ptosis, each occurring in 4% of patients. 

Most of the patients included in the review were managed with endovascular intervention via a transarterial approach, whereas only one patient underwent a transvenous procedure. Surgical management was employed in a smaller proportion of cases (n = 3), and only a single patient received conservative treatment.

Limitations

This case report is limited by the scarcity of documented CCF secondary to orbital stab wounds, which restricts meaningful comparison of treatment strategies and clinical outcomes. In addition, loss to follow-up related to the patient’s social vulnerability precluded long-term assessment of clinical evolution, functional recovery, and durable angiographic efficacy. Further limitations inherent to the literature review include publication bias, heterogeneous and inconsistent reporting of clinical, angiographic, and hemodynamic outcomes, and the paucity of standardized long-term follow-up data. Consequently, it was not possible to determine durable angiographic cure, the occurrence of late cranial neuropathies, or sustained functional recovery. These constraints limit the interpretation of the findings, as the effectiveness of staged endovascular approaches cannot be generalized from heterogeneous case reports, and reported outcome rates are likely underestimated or biased. Moreover, many of the reviewed cases lacked explicit angiographic documentation sufficient to allow precise classification of CCF, thereby limiting data reproducibility and hindering data homogenization across studies.

Future research

Future research should focus on the development of standardized reporting frameworks for traumatic CCF, including uniform documentation of injury mechanisms, angiographic characteristics, venous drainage patterns, treatment strategies, and short- and long-term clinical outcomes. Prospective data collection and multicenter collaboration may help overcome the limitations imposed by the rarity of these lesions, allowing more meaningful comparison of therapeutic approaches and staged endovascular techniques. In addition, longer and structured follow-up is essential to adequately assess durable angiographic cure, delayed cranial neuropathies, visual and functional recovery, and long-term vessel patency, thereby improving the quality of evidence and guiding future management strategies.

## Conclusions

CCFs following penetrating orbital trauma are rare but potentially vision-threatening lesions that require a high index of suspicion and early endovascular management. Visual prognosis is closely related to timely recognition; therefore, penetrating ocular injuries accompanied by persistent proptosis, chemosis, ophthalmoplegia, or orbital bruits should prompt early vascular imaging. This case underscores how patient vulnerability and loss to follow-up can delay diagnosis and treatment, ultimately resulting in poor visual outcomes.
